# Life Cycle and Transmission of *Cyclospora cayetanensis*: Knowns and Unknowns

**DOI:** 10.3390/microorganisms10010118

**Published:** 2022-01-06

**Authors:** Jitender P. Dubey, Asis Khan, Benjamin M. Rosenthal

**Affiliations:** Animal Parasitic Disease Laboratory, Beltsville Agricultural Research Center, Agricultural Research Service, United States Department of Agriculture, Building 1001, BARC-East, Beltsville, MD 20705, USA; asis.khan@usda.gov (A.K.); benjamin.rosenthal@usda.gov (B.M.R.)

**Keywords:** *Cyclospora cayetanensis*, humans, life cycle, transmission, surrogate

## Abstract

Although infections with *Cyclospora cayetanensis* are prevalent worldwide, many aspects of this parasite’s life cycle and transmission remain unknown. Humans are the only known hosts of this parasite. Existing information on its endogenous development has been derived from histological examination of only a few biopsy specimens. Its asexual and sexual stages occur in biliary-intestinal epithelium. In histological sections, its stages are less than 10 μm, making definitive identification difficult. Asexual (schizonts) and sexual (gamonts) are located in epithelial cells. Male microgamonts have two flagella; female macrogametes contain wall-forming bodies. Oocysts are excreted in feces unsporulated. Sporulation occurs in the environment, but there are many unanswered questions concerning dissemination and survival of *C. cayetanensis* oocysts. Biologically and phylogenetically, *C. cayetanensis* closely resembles *Eimeria* spp. that parastize chickens; among them, *E. acervulina* most closely resembles *C. cayetanensis* in size. Here, we review known and unknown aspects of its life cycle and transmission and discuss the appropriateness of surrogates best capable of hastening progress in understanding its biology and developing mitigating strategies.

## 1. Introduction

Cyclosporiasis is a problem worldwide. Several recent papers review prevalence, clinical symptoms, diagnosis, epidemiology, and treatment of cyclosporiasis [1,2,3,4,5,6]. The objectives of this paper are to review known and unknown aspects of its life cycle and transmission, and to review the appropriateness of surrogates best capable of hastening progress in understanding its biology and developing mitigating strategies.

## 2. Etiology

Among at least 20 species in the genus *Cyclospora*, *Cyclospora cayetanensis* is the only species known to infect humans [7]. Other congeners infect animals ranging from arthropods to non-human primates [8]. There are no in vitro or in vivo methods known for propagating *C. cayetanensis*; this severely limits development of new control strategies. Attempts to infect several species of laboratory animals, including non-human primates with *C. cayetanensis* were unsuccessful [9]. Information on its endogenous developmental stages came from histological examination of limited biopsy material of digestive tissues [10,11]. Considering these limitations, we discuss here taxonomic, phylogenetic, molecular relationships of *C. cayetanensis* with other closely related organisms suitable as surrogates to explore its biology and control.

## 3. Taxonomy

*Cyclospora cayetanensis* is classified as a coccidian parasite, in the phylum Apicomplexa, family Eimeriidae. All members of Eimeriidae have a one-host, fecal-oral cycle. *Eimeria* species are among the most prevalent parasites of livestock and poultry; infections are so prevalent that it is very difficult to raise livestock free of *Eimeria* [12]. *Eimeria* are host-specific, and infections are generally confined to gastro-intestinal system. Indeed, *Eimeria* species causing poultry coccidiosis impose high costs to poultry production, worldwide. As with *Cyclospora* spp., oocysts of *Eimeria* are excreted in feces unsporulated (non-infectious), and sporulation occurs outside in the environment. Although *Eimeria* oocysts (encasing two sporocysts, each with four sporozoites) morphologically differ from those of *Cyclospora* (encasing two sporocysts, each with two sporozoites), they are phylogenetically related [13]. *Cyclospora* comprise a monophyletic group which share common ancestor with all species of *Eimeria*, to the exclusion of other extant taxa [13].

## 4. Life Cycle of *C. cayetanensis*

**Figure 1:**
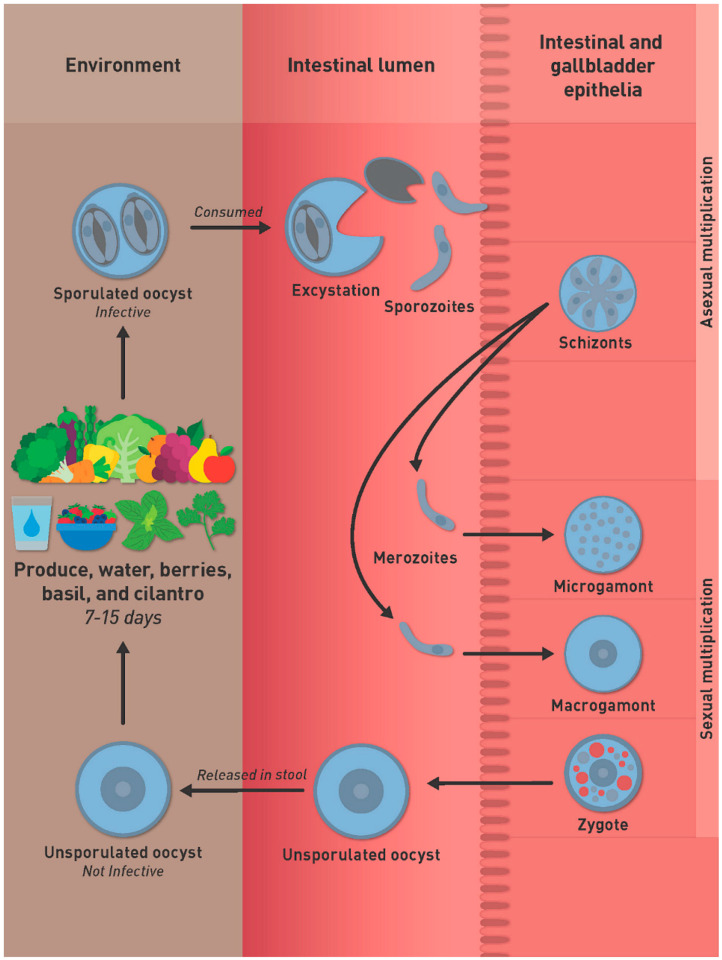
Proposed life cycle of *Cyclospora cayetanensis*.

The life cycle of *C*. *cayetanensis* remains incompletely defined. The best-characterized stage is the oocyst. Humans are thought to be infected by ingesting food or water contaminated with oocysts (Figure 1).

If a susceptible human ingests sporulated oocysts in contaminated food or water, the sporozoites inside the sporocysts excyst in the gut lumen and invade intestinal-biliary epithelial cells where the sporozoites transform into schizonts. Sexual multiplication occurs at the same site as asexual cycle. Microgamonts (male) contain flagellated microgemetes that fertilize macrogamonts to form the zygote. Oocysts then are excreted unsporulated in the feces. The prepatent period is thought to be around one week. Unsporulated oocysts are not infectious, they need to sporulate to became infective for a host. Under laboratory conditions, at 22 °C and 30 °C, sporulation will take around 7–14 days to occur outside the host [6].

### 4.1. Oocysts

The original description of *C*. *cayetanensis* elucidated most of the features presently understood about its oocysts [7]. Unsporulated oocysts in fresh fecal smears are spheroidal, 8–10 µm in diameter, with little or no size variation. The oocyst wall is colorless, thin (<1 µm), and bilayered. The inner layer encloses a central, undivided mass called the sporont; the sporont almost fills the oocyst and has many globules [7]. Sporulation occurs outside the host. Polar body and oocyst residuum are present. In fully-sporulated oocysts, there are two sporocysts, each with two sporozoites. Sporocysts are ovoidal, ~4 × 6 µm, and contain Stieda and substieda bodies, and a large residuum. The Stieda bodies are plug-like structures involved in sporozoite excystation [12]. The sporozoites are elongated, ~1 × 9 µm [7]. Whether *C. cayetanensis* sporozoites have crystalloid body or refractile bodies is not known. In *Eimeria* species, crystalloid bodies are considered virus-like particles; refractile bodies are proteinaceous in nature and are linked to host cell invasion [12].

### 4.2. Asexual Multiplication

After people ingest sporulated oocysts, sporozoites are believed (based on by analogy to more detailed data from various species of *Eimeria*) to excyst in the gut lumen and multiply, asexually, in intestinal epithelial cells. A few sporozoites may spill over and invade bile ducts and the gallbladder. Based on histological examination of endoscopic biopsies of duodenum or upper jejunum of Peruvian patients, asexual stages were observed in intestinal epithelial cells (presumably enterocytes) [10]. Only a few parasites were observed, and thus results were pioneering but preliminary. These stages were termed Types I and II meronts. Type I meronts each contained 8–12 small (3–4 μm long) merozoites; Type II meronts each contained four (12–15 μm) merozoites. However, the size of these merozoites was not confirmed by others [11]. The mode of division was not described.

**Figure 2:**
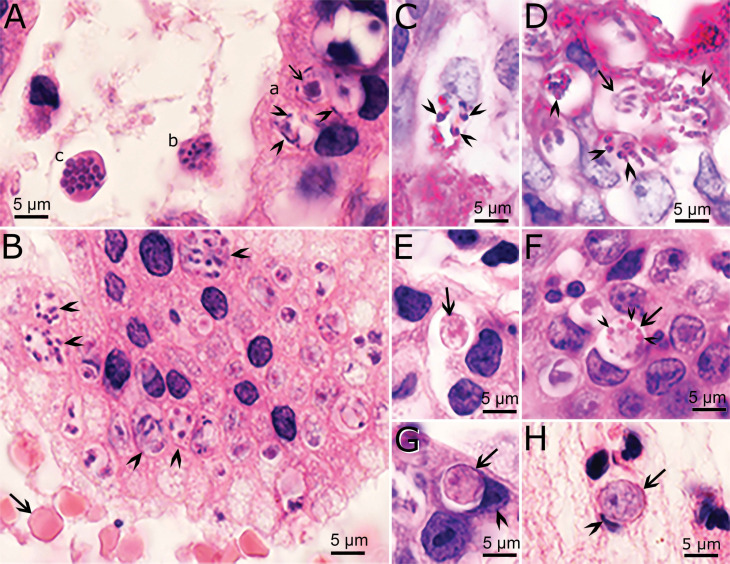
Stages of *Cyclospora cayetanensis* in the gallbladder of an immunosuppressed patient. (**A**,**B**,**E**–**H**) Hematoxylin and eosin stain. (**C**,**D**) Periodic acid Schiff (PAS) reaction, counter-stained with hematoxylin. Bar applies to all figures. (**A**) The glandular epithelium is on the right and lumen on the left. (a) Three microgamonts in epithelial cells. Note peripherally located microgametes (arrowheads) around a large residual body (arrow). (b, c) Schizonts free in the lumen of a gland. Note cross section (c) of merozoites in a schizont. (**B**) Desquamated epithelium in lumen of a gland. Note numerous schizonts (arrowheads) and red blood cells (arrow) for size comparison. (**C**) A schizont with PAS-positive (arrowheads) merozoites. (**D**) Several schizonts with variable PAS-positive granules (arrowheads). Arrow points to a PAS-negative schizont that has merozoites attached to a residual body (arrow). (**E**) An early macrogamont (arrow). (**F**) A late macrogamont (arrow) with eosinophilic wall-forming bodies (arrowheads). The host cell nucleus is indented. (**G**) An intracellular oocyst (arrow) in the epithelium. Note indented host cell nucleus (arrowhead). (**H**) An oocyst (arrow) free in lumen of gallbladder. Arrowhead points to indented host cell nucleus [11].

Recently, an opportunity arose to study endogenous development of *C*. *cayetanensis* in an immunosuppressed cyclosporiasis patient with severe abdominal symptoms [11]. The entire gallbladder from this case had been surgically removed and fixed for histological and transmission electron microscopic (TEM) examination. In this gallbladder, profuse parasitization was observed; the diagnosis was confirmed by the presence of *C*. *cayetanensis* oocysts in feces and PCR, using DNA extracted from the gallbladder [11]. *Cyclospora cayetanensis* stages were found only in epithelial cells, located in a parasitophorous vacuole of host cytoplasm (Figure 2). Mature schizonts were 7.6 × 5.1 μm and contained up to 10 merozoites. The size of merozoites could not be determined but appeared to be less than 6 μm long. By TEM examination, schizonts divided by schizogony (where the parasite nucleus divided into five or more nuclei before segregating into distinct merozoites). A residual body was present in some, but not in others. The number of generations of schizonts could not be determined because of overcrowding. By light microscopy, merozoites and schizonts in different phases of development appeared within the same host cell or in adjacent host cells. Whether the course of schizont development in this case was influenced by the immunosuppressive status of the patient could not be ascertained. Thus, caution is warranted before extrapolating; future studies should determine whether such asynchronous development occurs in immunocompetent patients. We are aware of no archived biopsy material available to support such studies.

#### Asexual Multiplication in Related Coccidia

Details of the asexual division process can aid diagnosis and illustrate the close affinity of *C*. *cayetanensis* with certain other coccidia. Considerable confusion surrounds the terminology used to describe asexual stages of coccidia. For the benefit of researchers not familiar with developmental cycles of coccidia, a brief account is provided here. Meront and schizont each describe stages undergoing asexual division. “Meront” has been used to denote various asexual stages, irrespective of division status [12]. Thus, it lacks perfect specificity. Some coccidia (for example, *Toxoplasma gondii*) replicate by “endodyogeny”, wherein each mother cell produces, and is consumed by, exactly two daughter cells. Others (for example, species of *Sarcocystis*) divide by endopolygeny, where the mother cell’s nucleus becomes a mass of connected lobes before subdividing into many daughter cells [12]. 

In species of *Eimeria*, schizonts divide by schizogony. After their sporozoites invade the host cell, the parasite nucleus divides into four or more nuclei before any merozoites are formed. After a defined number of multiplication cycles (termed generations), characteristic of each species of *Eimeria*, parasites from the final merozoite generation initiate the sexual cycle. The number of generations is fixed for each *Eimeria* species, and is not altered by immunosuppression [12]. Merozoites from each generation of schizonts are morphologically distinct. 

Schizogony in *C*. *cayetanensis* resembles that in *Eimeria*. The lack of sequential specimens for study has, to date, precluded determination of the number of generations it undergoes prior to initiating the sexual cycle. Nonetheless, the occurrence of merozoites of different sizes suggests the existence of multiple generations of schizonts [12]. The development of merozoites by schizogony was confirmed by TEM examination. The presence of Type II merozoites could not be confirmed.

**Figure 3:**
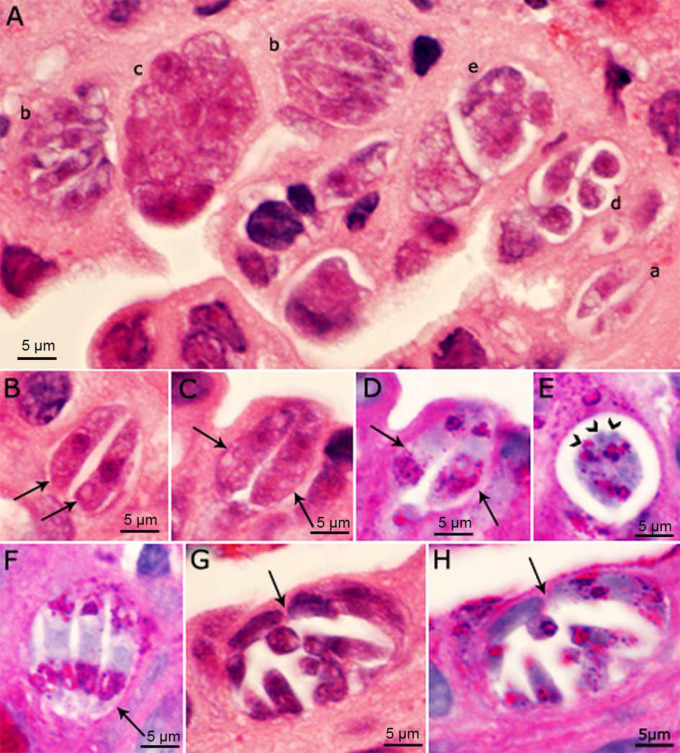
Asexual stages of *Cystoisospora belli* in histological sections of bile duct of a patient. Bar applies to all parts. (**A**–**C**,**G**) = Hematoxylin and eosin stain; (**D**–**F**,**H**) = PAS counter stained with hematoxylin. The luminal side of sections is on the top. (**A**) Several meronts in one microscopic field. (a) Paired merozoites in one pv. (b) Meront with four or more merozoites completely filling the pv. (c) Meront containing developing merozoites. The organisms are cut in cross-section; the nuclei appear larger than in merozoites. (d) Four merozoites in a pv with spaces between merozoites. (e) Immature meront. (**B**) Paired crescent-shaped merozoites. (**C**,**D**) The same paired organisms/meronts after staining with HE (**C**) and PAS (**D**). Note the polar PAS-positivity. The parasite nuclei are masked by PAS staining. (**E**) Meront with mero- zoites budding (arrowheads). Note PAS-positivity. (**F**) Meront (arrow) with three merozoites with polar PAS-positivity. (**G**,**H**) The same mature meront (arrow) after staining with HE (**G**) and PASH (**H**) [14].

*Cystoisospora belli* is another coccidian that also parasitizes the small intestine and biliary system in humans [14,15]. In *Cystoisospora*, different generations of schizonts/Types can be found in the same host cell, because schizonts can rupture inside the host cell and merozoites can multiply within the same host cell without exiting the host cell (Figure 3). Additionally, immature schizonts can retain the shape of merozoites (Figure 3). Whether this type of multiplication also occurs in *C*. *cayetanensis* remains to be determined. Because *C. belli* stages (including merozoites, gamonts and oocysts) are more than twice the size of their counterparts in *C. cayetanensis,* differential diagnosis is possible to the trained eye using light microscopy [15]. Genetic assays also support differential diagnosis [16,17,18].

### 4.3. Sexual Stages

Sexual stages of *C. cayetanensis* are smaller than 10 µm and occur in the same location as schizonts [11]. The male microgamonts measure 6.6 × 5.2 µm and contain fewer than 20 microgametes organized around a residual body. Each microgamete measures up to 2 µm long and is flagellated. Female macrogametes contain distinctive eosinophilic wall-forming bodies that vary in size and are less than 1 µm in HE-stained sections. Macrogamonts measure 5.8–6.5 × 5.3–6.5 µm. Oocysts in sections are unsporulated and have a diameter of 5.7–7.5 µm. The TEM examination confirmed the histologic findings [11].

## 5. Need for Surrogates for Studying *C. cayetanensis*

Only rarely have scientists been positioned to directly study *C. cayatanensis* infections in vivo. Even access to oocysts is limited, undermining efforts to understand their maturation and senescence. What we know of human pathology and the parasite’s development in human hosts has been derived from just a few case studies. Ethical constraints essentially preclude the ability to examine infections under defined or replicated conditions, and the absence of any in vitro or animal model for studying this organism further impede progress in identifying vulnerabilities that might be exploited to strengthen prevention or cure. 

Fortunately, a wealth of data from natural and experimental *Eimeria* infections provide helpful context for interpreting what scant evidence we do have (see above). Furthermore, *Eimeria* can and should be exploited as surrogates for evaluating methods requiring large numbers of *C. cayetanensis* oocysts. For example, maturation of oocysts of *Eimeria acervulina* (a common poultry parasite) entails upregulation of a suite of genes most of which have homologues in *C. cayetanensis* [19]. These abundant parasites, which pose no risk to the health of human investigators, can speed efforts to evaluate methods to filter parasites from irrigation water, and treat food in ways that may render such contaminants harmless, or treat infections when prevention fails.

## 6. Molecular Tools and Comparative Genomics

Below, we briefly review insights from comparative genetics and genomics of *Cyclospora* illustrating the potential, and limits, of related organisms as surrogates to inform us of opportunities to better understand, and manage, this elusive but important human enteric pathogen [19,20,21,22,23,24,25,26,27,28,29,30,31,32,33,34,35,36,37,38,39,40,41,42,43,44].

### 6.1. Small-Subunit rDNA Sequence

**Figure 4:**
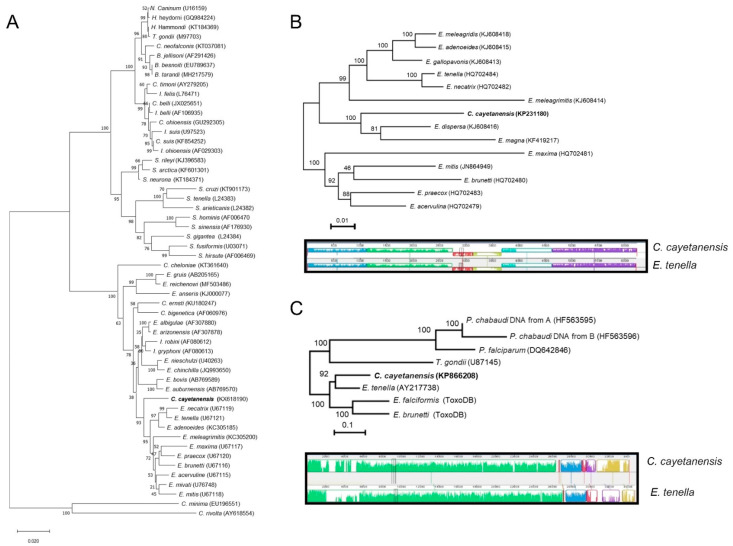
Phylogenetic relationship between *C. cayetanensis* and *Eimeria* species. Maximum likelihood phylogenetic tresses were constructed using (**A**) 18S rDNA, (**B**) mitochondrial genome, and (**C**) apicoplast genome. Synteny in genome organizations between *Cyclospora cayetanensis* and *E. tenella* is shown in the mitochondrial (**B**) and apicoplast (**C**) genomes. (**B**,**C**) were modified with permission from Refs. [30,34].

Molecular tools have revolutionized the exploration of biodiversity. Sequencing nuclear-encoded small-subunit rDNA gene (18S) has especially aided reconstruction of relationships among organisms for which morphological taxonomy is challenging [20]. Comparisons of SSU-rRNA sequences between human and primate isolates of *Cyclospora* spp. identified 98% similarity in that gene [21], validating their distinctness but testifying to their close evolutionary relationship. SSU-rDNA was also utilized to understand the relatedness among closely related species. Strikingly, examination of SSU-rRNA sequences revealed a 94 to 98% sequence similarity between *Cyclospora* and *Eimeria* species. The neighbor-joining tree constructed using 18S rDNA sequences suggests *C. cayetanensis* is most closely related to *Eimeria* species that infect birds (Figure 4A). Thus, molecular analyses of nuclear SSU rDNA sequences suggest that *C. cayetanensis* is closely related to other coccidia, especially members of the genus *Eimeria* in the family Eimeriidae.

This molecule serves as the target for FDA-validated assays for diagnosing contamination of certain types of fresh produce with *C. cayetanensis* from fresh produce and is also used to diagnose infections in clinical samples [22,23,24,25]. The sensitivity of assays targeting this gene benefits from the fact that each genome of the parasite encodes multiple copies of the SSU rRNA. Recently, a real-time qPCR assay targeting the *C. cayetanensis* 18S rDNA gene has been optimized to improve detection sensitivity, as validated in the FDA Bacteriological Analytical Manual (BAM) for *C. cayetanensis* testing [24]. 

### 6.2. Organellar Genomes

Organellar genomes, residing in the mitochondrion and apicoplast, illuminate relatedness among closely related apicomplexan species because they are inherited maternally without genetic recombination [26,27]. Recently, the mitochondrial and apicoplast genomes of *C. cayetanensis* have been sequenced and compared with closely related *Eimeria* species [28,29,30,31]. As with other coccidian parasites, the mitochondrial genome of *C. cayetanensis* is 6229 bp in size with 33% GC content [28,29,30,31]. The mitochondrial genome is organized in a linear molecule with approximately 513 copies; these are arranged in concatemeric structures with a head-tail configuration, as in other *Eimeria* mitochondrial genomes. As with other coccidian parasites, the mitochondrial genome of *C. cayetanensis* encodes three protein-coding genes (*cox1*, *cox3*, and *cytB*), in addition to 14 large subunit (LSU) and nine small subunit (SSU) fragmented rRNA genes [30]. Comparing the amino acids comprising the mitochondrially-encoded proteins of *C. cayatanensis* to those of closely-related species of *Eimeria* documented sequence similarities ranging from 90–97% for *cytbB*, 93–97% for *cox1*, and 83–93% for *cox3* [30]. Additionally, comparative phylogenomic analysis of mitochondrial genomes also revealed the monophyletic clustering of *C. cayetanensis* with the rabbit-infecting parasite *E*. *magna* (Figure 4B) [30]. Comparing the mitochondrial genomes of isolates of *C. cayetanensis* identified 8 single nucleotide polymorphisms and one 7-bp multiple-nucleotide in the junction area between genome copies [28,32].

The apicoplast of *C. cayetanensis,* a non-photosynthetic plastid derived from a secondary symbiosis, is 34,155 bp in length, with a GC content of 22%, and harbors 29 protein-coding genes, 33 tRNA, and four rRNA genes [28,33]. Genes are encoded bidirectionally. A recent comparative study of sequence variation of apicoplast genomes of *C. cayetanensis* identified 25 single nucleotide differences amongst 11 isolates from several geographical locations, in addition to a unique 30 bp-long repeat insertion sequence in a Nepalese sample [28,33]. 

Comparing the apicoplast genome of *C. cayetanensis* to that of *Eimeria tenella* (Table 1) revealed complete conservation of gene order (synteny) and strong (85.6%) sequence conservation (Figure 4C) [28]. Thus, the genetic similarity between *Eimeria* species and *C. cayetanensis* in each organellar genomes substantiate avian *Eimeria* as a sensible surrogate to study drug treatment, and therapeutics specifically targeting the organellar genomes.

### 6.3. Nuclear Genome

Currently, 39 nuclear genomes of *C. cayetanensis* have been assembled, using either short paired-end reads from Illumina sequencing (Illumina, San Diego, CA, USA) or Roche GS-FLX 454 sequencing (454 Life Sciences, Branford, CT, USA, available online at https://www.ncbi.nlm.nih.gov/assembly/?term=cyclospora, accessed on 15 November 2021) [34,35]. Of these 39 genomes, CcayRef3 (GCF_002999335) and CHN_HEN01 (GCF_000769155) presently serve as the reference genomes of *C. cayetanensis*. The total nuclear genome size is estimated to be ~44 Mb (Table 1) [34,35]. These genomes have roughly similar GC composition (52%) and are predicted to encode ~7500 genes; they have been resolved to 738 contigs (Table 1). Not surprisingly, comparative genome analysis revealed that the genome organization, metabolic capabilities, and potential invasion mechanisms are very similar to those of *E. tenella* [34]. When compared with more distantly related relatives that form tissue-cysts (such as *T. gondii*), these monoxenous parasites are notably reduced in rhoptry protein kinases, phosphatases, and serine protease inhibitors. This seems significant because of the dominance of such protein domains in tissue-cyst forming coccidian parasites; this underscores the special suitability of *Eimeria* as surrogates to study the enigmatic *Cyclospora* spp. Note, however, that even these close relatives differ, reminding us of the uniqueness of each biological species: a family of TA4-type surface antigens (SAG), highly abundant in *E. tenella*, have not been detected in *C. cayetanensis* [34]. In addition, plant-like AP2 transcription factors are slightly more abundant in *C. cayetanensis* than in *E. tenella*, consistent with these being the major transcription factors in apicomplexans [34].

### 6.4. Developmentally-Regulated Genes

Genome assembly and annotation for *Cyclospora* and for its *Eimeria* relatives remain far from complete, impeding efforts to understand their comparative biology. Nonetheless, a recent study of sporulation in *E. acervulina* suggests that similar genes in each species may be important regulators or effectors of parasite maturation [19]. Maturing cohorts of *E. acervulina* undergo concerted changes in the expression of many genes; most of these increase their expression over the course of sporulation. Genes strongly expressed throughout sporulation included ATPases (like EAH 4100 and EAH 4110) and oocyst wall proteins (like EAH 33530). Members assigned to the SAG family of surface antigens (EAH 59200, 59950, and 11680) were expressed to a much greater extent in mature than in immature oocysts. Although the function of many other genes remains unknown, guided at best by predictions from gene ontology, it is notable that about two-thirds of these genes have strongly conserved genes in *C. cayetanensis.* Until it becomes possible to propagate and study *C. cayetanensis* (in animal models, in vitro, or in organoid cultures), *Eimeria* surrogates offer a biologically sensible means to experiment with parasites, to define their biology, tolerances, and vulnerabilities. 

## 7. Conclusions

To counter the harms posed by *C. cayetanensis,* producers, public health authorities, clinicians and researchers require more empirical studies of this enigmatic parasite. However, the episodic nature of its transmission and the absence of in vitro or in vivo means to propagate the parasite severely limit such opportunities. Fortunately, a wealth of information exists for the veterinary parasites which *Cyclospora* most closely resembles. These similarities span transmission, development, genetics, pathogenesis, and molecular biology. The experimental opportunities afforded by these readily studied surrogates should hasten progress in understanding, and better managing, threats to food safety and public health.

## Figures and Tables

**Table 1:** Comparison of genomic features of apicomplexan parasites.

Category	*Cryptosporidium parvum*	*Plasmodium falciparum*	*Babesia bovis*	*Sarcocystis neurona*	*Neospora caninum*	*Hammondia hammondi*	*Toxoplasma gondii*	*Eimeria tenella*	*Cyclospora cayetanensis*
Reference	[34,36]	[34,37]	[34,38]	[39,40]	[39,41,42]	[39]	[39,42,43]	[34,44]	[34,35]
No. of chromosomes	8	14	4	NA	13	14	13	14	NA
Estimated size (Mb)	~9	~23	8	~127	~62	~65	~65	52	44
GC content (%)	30.3	19.4	41.8	51.5	54.8	52.5	52.2	52.5	51.8
No. of protein-coding genes	3897	5542	3671	7093	7540	8004	8322	8597	7457
Mean length of protein-coding genes (bp)	1799	2271	1514	9121	4872	4868	4778	1518	1599
Apicoplast genome size (kb)	NA	~34	~33	~35	~35	~35	~35	~35	~34
Mitochondrial genome size (bp)	NA	~6	~6	NA	~6	~6	~6	~6	~6

NA, not applicable.

## Data Availability

Not applicable.
